# Facilitators and Barriers to Healthy Eating in Aged Chinese Canadians with Hypertension: A Qualitative Exploration

**DOI:** 10.3390/nu11010111

**Published:** 2019-01-08

**Authors:** Ping Zou

**Affiliations:** School of Nursing, Nipissing University, 750 Dundas Street West, Room 209, Toronto, ON M6J 3S3, Canada; pingz@nipissingu.ca; Tel.: +1-416-642-7003

**Keywords:** diet, facilitators, barriers, healthy eating, immigrant, Chinese, Canada

## Abstract

**Objectives**: To determine the facilitators and barriers influencing healthy eating behaviours among aged Chinese-Canadians with hypertension. **Methods**: After attending five weeks of dietary educational training (Dietary Approach to Stop Hypertension with Sodium (Na) Reduction for Chinese Canadians; DASHNa-CC), 30 aged Chinese-Canadian participants partook in a telephone interview. Participants were asked to name three facilitators and three barriers that influenced their ability to follow the DASHNa-CC intervention. Telephone transcripts were then analyzed and coded using computer software and categorized into personal, familial, community, and societal facilitators or barriers. **Results**: Personal factors included health problems, motivation, effects of healthy diet, health-related careers, and dietary habits. Family factors included family structure, support from family members, and critical health events involving family members or relatives. Community factors consisted of educational materials, friends, primary care physicians, and online social networks. Societal factors included accessibility to grocery stores and restaurants. **Conclusions**: Aged Chinese-Canadian immigrants experience unique facilitators of and barriers to healthy eating, which may warrant further attention from healthcare professionals when educating patients in a culturally-sensitive manner.

## 1. Introduction

In Canada, despite the advancement of technology and medical science, the hypertension prevalence rate and uncontrolled rate remain high [1]. Hypertension costs the Canadian health care system an estimated $2.4 billion each year in hospitals, physician visits, prescription medications, and special treatments [2]. In Canada, 1.3 million Chinese citizens comprise approximately 4.0% of Canada’s population and 21.1% of the country’s visible minorities [3]. With a 15.1% hypertension prevalence rate [4], Chinese Canadians are at high risk of cardiovascular diseases and associated morbidity and mortality. Diet has been identified as the most important risk factor for hypertension in the Chinese population [5]. There is an urgent need for understanding Chinese-Canadians’ dietary behaviors and planning effective dietary interventions for blood pressure control [6].

The typical Chinese diet is characterized by its high sodium, low potassium, and low calcium intake [5]. According to the International Study of Macro/Micro-Nutrients and Blood Pressure (INTERMAP), Chinese individuals had the highest 24-hour urinary sodium excretion, the lowest urinary potassium excretion and the highest urinary sodium/potassium ratio, and the lowest dietary calcium intake compared to their counterparts in the United States, United Kingdom, and Japan [7]. According to the latest national survey of nutrition and health status, Chinese citizens reported that a mean daily salt intake of 12 g [8], which significantly exceeds the Canadian Hypertension Education Program’s (CHEP) sodium recommendation of 3.0 to 3.8 g [9]. In the Hypertension in the Very Elderly Trial, Chinese participants with hypertension had a significantly lower serum potassium concentration than their counterparts recruited from other countries (4.25 mmol/L vs. 4.42 mmol/L in men and 4.26 mmol/L vs. 4.38 mmol/L in women; *p* < 0.05 for both men and women) [10]. In addition, Chinese Canadians tend to have a low intake of calcium. Yu et al. (2012) reported that the risk for calcium inadequacy was 36% for males and 78% for females in adult ethnic Chinese participants (*n* = 81) in Alberta, Canada [11]. These findings coincided with another community survey in the United States (*n* = 399), which indicated that Chinese individuals in North America consume dairy products only monthly, with the exception of milk powder [12].

Chinese dietary behaviors of high sodium, low potassium, and low calcium intake are the most important risk factors for hypertension [5], as they are clinically implicated in the pathogenesis of hypertension. High sodium intake is associated with high nocturnal blood pressure and low nocturnal fall of blood pressure [5]. A study in Hong Kong (*n* = 111) assessed dietary sodium, potassium, and calcium intake by 24-hour diet recall, and found that those with hypertension had a lower calcium intake (411 +/− 324 vs. 589 +/− 428 mg, *p* = 0.04) and a higher sodium/potassium ratio (4.7 +/− 2.8 vs. 3.4 +/− 2.3, *p* = 0.02) compared to controls. Participants with high urinary sodium/potassium ratios and low calcium intake had significantly higher systolic blood pressure measurements (159 +/− 26 vs. 130 +/− 15 mmHg) and higher prevalence of hypertension (78% vs. 25%) compared to controls Systolic blood pressure was negatively correlated with calcium intake (*r* = −0.40, *p* < 0.05) and positively correlated with urinary sodium/potassium ratio (*r* = 0.30, *p* < 0.05) [13].

In response to high hypertension prevalence rates among Chinese Canadians, the Dietary Approach to Stop Hypertension with Sodium (Na) Reduction for Chinese Canadians (DASHNa-CC) intervention was designed to provide a standardized, culturally sensitive dietary education platform for hypertension control [14]. The findings of the DASHNa-CC pilot trial suggested that participants accepted the intervention well and improved their dietary behaviours when comparing pre- and post-intervention data. In addition, the DASHNa-CC intervention may have the potential to decrease blood pressure and improve health-related quality of life for Chinese Canadians. However, it is unknown which factors promote or prevent from healthy eating behaviours. Without an understanding of the key personal, community and societal factors contributing to Chinese Canadians’ dietary behaviours, it will be difficult to adopt the DASHNa-CC intervention in different settings with assurance of patient satisfaction and intervention effectiveness. As a sub-study of the DASHNa-CC, the current study proposed research questions as follows: (a) What are the facilitators promoting healthy eating behaviours among aged Chinese Canadians with hypertension? (b) What are the barriers preventing from healthy eating behaviours among aged Chinese Canadians with hypertension?

In this paper, the ecosocial theory is used to guide data collection, data analysis and presentation of the findings. The ecosocial theory is a broad and complex theoretical framework to explore the relationship between illness distribution and its social determinants [15]. While the theory emphasizes psychosocial influences on disease occurrence, the theory is suited to analyze the relationships between social factors and disease development in community. The use of ecosocial theory promotes understanding of comprehensive factors influencing an illness distribution and encourage critical thinking on creative approach for appropriate intervention for health and wellness.

## 2. Methods

Following university ethics approval, participants were recruited from the DASHNa-CC study participants. The study design, main outcome and recruitment process of the DASHNa-CC study has been published [14,16]. After receiving 5 weeks of the DASHNa-CC intervention, all participants in the intervention group consented to receive a 30 to 45 min telephone interview. The telephone interview was delivered by the author at the date and time chosen by the participants. While the telephone interviews were audio recorded, the Telephone Interview Form was also used to document the communications during the interviews. The participants were asked two open-ended questions over the phone, to be specific, (a) to identify three most important factors that helped them follow the recommendations of the DASHNa-CC intervention; and (b) to identify three most important factors that prevented them from following the recommendations of the DASHNa-CC intervention.

Using NVIVO 11 software, thematic analyses were conducted. Data from interviews were transcribed into word processing files. The transcripts were analyzed by generating a list of themes and codes to provide evidence reflective of broader perspectives. Open and axial coding were applied to the data [17]. During open coding, each transcript was reviewed by the author and the research assistant, and the data were reduced to codes. Differences in coding were resolved during discussions. Codes that were found to be conceptually similar in nature or related in meaning were grouped into subcategories. In axial coding, the intent was to clarify how the emergent subcategories were related to preliminary categories. Analytical tools, including asking questions and making comparisons, were utilized to find the properties of each concept. Establishing both credibility and reliability is crucial when employing qualitative methods [18,19,20]. Several strategies including substantial engagement [18], progressive subjectivity [21,22], member checks [23], and triangulation [20,24] were applied.

## 3. Results

### 3.1. Participant Characteristics

Participants of this study were aged Chinese Canadians (*n* = 30) with stage one hypertension in the intervention group of the DASHNa-CC study. At the baseline, the mean systolic blood pressure of participants was 145.6 mmHg (SD = 11.1) and the mean diastolic blood pressure was 90.5 mmHg (SD = 7.5). 16 women and 14 men were included in this study. The mean age was 60.8 years (*SD* = 11.7) and the mean number of years living in Canada was 9.7 (*SD* = 5.7). The large proportion of participants lived a southern Chinese lifestyle (*n* = 20, 66.7%), held a bachelor degree (*n* = 13, 43.3%), were retired (*n* = 15, 50%), were married (*n* = 25, 83.3%), had an annual family income less than $20,000 (*n* = 16, 53.3%), and had a family history of hypertension (*n* = 13, 43.3%). The mean waist circumference was 87.3 cm (*SD* = 5.9) for women and 91.2 cm (*SD* = 5.3) for men (see Table 1).

**Figure 1:**
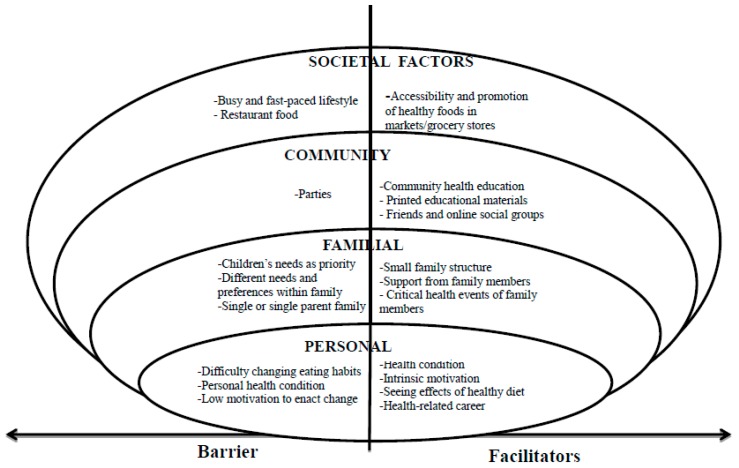
Facilitators of and Barriers to Healthy Eating.

Guided by the ecosocial theory, the facilitators and barriers to healthy eating experienced by the aged Chinese Canadian immigrants in this study were grouped within the following four categories: (a) personal factors, (b) familial factors, (c) community factors, and (d) societal factors (see Figure 1).

### 3.2. Personal Factors

Personal factors that facilitated healthy eating behaviours amongst the participants included: (a) having a health condition, (b) having intrinsic motivation to eat well, (c) experiencing positive effects of healthy diet, and (d) having a job in healthcare.

Participants who had a health condition that could be improved through healthy eating reported the desire to change their eating behaviours for the sake of their health status. As one participant explained, “Currently, I am unhealthy. I must change my eating habits in order to properly address my health needs. I worry about my health, so I implement measures consciously (ID013C)”. Other participants also acknowledged that their poor health status is what motivated them to seek the knowledge needed to enact healthy eating (ID820C, ID984D, ID013B, ID020C).

In addition to motivation from poor health status, some individuals possessed intrinsic motivation to engage in healthy eating. As one participant shared, “I have the motivation to eat well. Healthy food is beneficial to health, so why not do it (ID020A)?” Several participants attributed their intrinsic motivation to eat healthy to the fact that (a) they understand the importance of maintaining good health, (b) they believe that diet influences health, and (c) they have the desire to live a long life (ID986C, ID046D, ID081A, ID016A, ID018C, ID859D).

Individuals who were able to see concrete effects of healthy eating expressed increased motivation to continue with changed eating behaviours. One participant expressed, “If I see the effect, I will have more confidence in healthy eating to control high blood pressure (ID027D)”. This was echoed in another participant’s statement, expressing, “I measure my blood pressure frequently. After I saw the effect of food treatment, I have more confidence [in healthy eating] (ID859E)”.

Participants who were health care providers generally reported to engage in healthier eating behaviours. As one participant explained, “My family and I are all medical professionals and we conduct research. We are aware of these principles and understand the importance of healthy food (ID858B)”. Health care providers also reported that their healthy eating patterns positively impacted the behaviors of other family members. A participant explained this phenomenon as follows, “My [healthy] eating habits affect my family members subtly. My husband loved to eat high salt, high sugar, and high cholesterol foods before. Now he has made progress and eats brown rice and brown bread too (ID324G)”.

Personal factors that acted as barriers to healthy eating included: (a) difficulty changing tradition, (b) having a personal health condition, and (c) low intrinsic motivation, especially when an individual is healthy.

Participants reported that it is difficult to change eating habits acquired over a lifetime. Participants stated that the process of changing eating behaviours is time consuming and is a test of willpower (ID987A, ID865A, ID820A, ID069H, ID046F, ID018G, ID865G). Even when aware of the health benefits of changing eating behaviours, it can be extremely difficult to do. As one participant shared, “It is very difficult to change 40 to 50 years of eating habits (ID987A)”. A specific element of difficulty was that eating healthier equated to eating less flavorful food (ID018G). For instance, a participant commented, “I always want to have delicious food but delicious food usually has more oil, more salt and more sugar. It is difficult to keep a balance between health and delicious food (ID865B)”.

Some individuals with a health condition expressed that their condition was a barrier to healthy eating. These individuals, some of which had specific food intolerances, explained that adapting their diet to specific food guidelines to facilitate healthier eating was challenging because they are limited to the foods they can eat. For example, lactose intolerance was a health condition constantly cited as a barrier to healthy eating. One participant explained, “Dairy products are hard for me to digest. It is difficult for me to consume certain food (ID299A)”.

Finally, participants reported that they lack motivation to make diet changes when they perceive themselves as healthy individuals. One participant shared, “Self-motivation is not enough. When our body functions normal, we don’t think about being sick. Therefore, sometimes we are not very strict about food. If there is a real, severe illness, then having healthy food is like taking medication. We will pay much more attention to food intake in the event of an illness (ID069C)”.

### 3.3. Familial Factors

Familial factors that facilitated healthy eating behaviours included: (a) small family structure, (b) support from family members, and (c) critical health events of family members.

Individuals who lived with their partners or lived alone reported that small family structure facilitated their control of their diet. As one participant who lived with their partner expressed, “It is easy to control [diet] within a small family because there are only two people having meals together (ID299B)”. Another participant stated, “both husband and wife get involved in eating healthy and there are no external influences. We attended the workshop together and felt it is very useful. Both of us want to change and know that we should change our bad eating habits (ID987B)”. Some participants appreciated living alone as a facilitator (ID046F, ID081D, ID363B). For instants, a participant stressed the sense of control within her single family unit, “I live by myself. No one disturbs me and it is easy to control my diet (ID801D)”.

Direct support from family members played an important role in facilitating healthy eating behaviours. Many of the participant comments focused on the positive influence when both husband and wife tried to improve their eating behaviours together (ID950A, ID016B, ID020B, ID069E, ID859C, ID013A, ID987B, ID982D, ID013A, ID987B). One participant explained, “My wife’s support is my motivation. My wife is very strict on diet and I follow what she does. For example, I add salt using a small spoon (ID822C)”. Some participants also valued the support their partners provided by attending community health workshops together (ID987B, ID827F).

When an immediate family member or relative experienced a critical health event, participants reported paying more attention to their own eating behaviours to improve their health and prevent a related adverse critical event from happening to them. One participant stated, “It was very shocking for me that my relative died from a stroke. The relative had high blood pressure, but he didn’t take the condition seriously. He had a stroke and died (ID026F)”.

Familial factors that acted as barriers to healthy eating behaviours included: (a) prioritizing children’s wants, (b) succumbing to different preferences within family, and (c) having a unique family structure (living alone or as a single parent).

Individuals with children reported to prioritize their children’s desires when selecting food and cooking. Parents commented that their children often preferred to eat oily foods, meat and foods that are full of flavour (ID291A, ID294A, ID859B). One parent elaborated, “We need to consider the children’s eating habits when cooking food … We have to cook what they like (ID069A)”.

Similarly, when family members (particularly in big families) had different preferences or priorities in food, participants experienced barriers to healthy eating. Participants expressed feeling a lack of control in what they ate when a particular family member did all of the cooking for the family. One participant stated, “It is difficult to reduce salt because my father-in-law does the cooking. If I cook by myself, I add less salt in the dish (ID942A)”. Participants also expressed the inability to cook independently in a big family, the difficulty of pleasing taste preferences across multiple generations that live together, the differences in perceived health needs and thus diet across family members, and the lack of support in eating healthy experienced in big families for if the food does not taste good, it will not be eaten (ID827C, ID986B, ID027A, ID046A, ID018A). As one participant summarized, “It is hard to satisfy everyone in a big family. We have to consider everyone’s eating habits. For example, my son likes to eat more meat and oily food. Considering everyone’s eating habits is the biggest barrier to healthy food (ID294A)”.

It was also expressed through participant interviews that living alone or as a single parent presented a barrier to healthy eating. Single parents explained that the immense responsibility of raising children that ideally is shared between two people is now put onto one person, which makes time limited. Thus, cooking and eating must be convenient. However, convenience does not often equate to healthy food options. One single parent expressed, “My husband will share in the house chores and help cook healthy food when he comes [to Canada] next year (ID859C)”. Additionally, a participant who lived alone explained, “I live by myself. I do not want to cook. I do not mind what kind of food I eat. Many people give me food to eat, so I eat a lot of high sugar, high salt, and high cholesterol food (ID829A)”.

### 3.4. Community Factors

Community factors that facilitated healthy eating behaviours included: (a) community health education workshops, (b) printed educational materials, and (c) friends/online social groups.

Participants stated that it was very useful to have workshops and training sessions pertaining to eating healthy within their community. Participants reported to gain valuable knowledge on making healthy dietary choices as a result of attending the workshops (ID822B, ID827E, ID986E, ID950B, ID027C, ID364A). Participants shared that they learned many strategies to make changes in their diet. For example, one participant explained, “It was helpful to attend the lecture …I knew that food with high calcium and high potassium had health benefits before, but I didn’t know what specific foods to choose. The problem was solved by attending the lecture (ID046E)”. The printed educational materials received at the workshops were also valued by participants and were reported to act as an ongoing reference material that supported participants in their journey to achieve healthier eating (ID982E, ID984A, ID013E, ID018F). One participant stated, “I put the manual on the table and always read the manual. I always review it and remind myself all the time (ID018F)”. Another participant also exclaimed, “The content of the manual is very good and the implementation is very concrete…by achieving nutritional balance, I feel good (ID081B)”.

The influence of like-minded friends and online social groups also helped individuals achieve a healthier diet. Individuals reported to be inspired to eat healthy by seeing negative health outcomes of friends who eat poorly and conversely, by being surrounded by friends who eat healthy and encourage them to do that same (ID858A, ID299C). As one participant shared, “Friends expect you to be healthier. My friends gather together to exchange ideas about nutrition. I feel I should be a good example (ID829C)”. Individuals reported to especially find value and inspiration from the support of their primary healthcare providers (e.g., traditional Chinese medicine practitioners and family physicians) (ID865E, ID984E). Social networking (e.g., WeChat) was also a communication tool used by participants to promote healthy eating (ID825D, ID829C).

The only community factor that was illuminated as a barrier to healthy eating was the influence of community gatherings, such as parties. Participants expressed that it is hard to meet the food requirements of all individuals at parties and thus healthy eating is often sacrificed (ID829A).

### 3.5. Societal Factors

A societal factor that facilitated healthy eating behaviours amongst participants included the accessibility and promotion of healthy foods in supermarkets/grocery stores. Participants felt that local markets provide a variety of food options to facilitate a healthy diet (ID294C, ID324F). As one participant proclaimed, “… the supermarket supply is the basis of a therapeutic diet. The food in the market is safe, healthy, and comes at a good price (ID324F)”. Another participant reported feeling that some grocery stores do a good job of offering a wide selection of healthy items, such as low-salt and salt-free items (ID294C).

Societal factors that acted as barriers to healthy eating included: (a) living a busy and fast-paced lifestyle and (b) frequently eating at restaurants.

Many participants repeatedly stated that they are too busy and have no time to cook, which makes healthy eating hard and negatively impacts the quality of the food they eat (ID827A, ID859A, ID363A, ID825A, ID324A). One participant stated, “I am very busy and have meetings frequently. Sometimes I only have half an hour to cook. I have no time to do what was suggested during the presentation (ID825A)”.

Individuals also explained the barriers that eating out for dinner presents in terms of selecting healthy options. Eating out for meals has become a societal norm, and as one participant described, “Most food in a restaurant is high in oil and salt. It is difficult to choose healthy food and we have no control when we eat out. Chinese restaurants need to change the cuisine and offer a healthy choice (ID018B)”. Participants reported it is very hard to control intake, especially salt, when eating at restaurants because of lack of availability of healthy items at restaurants (ID825E, ID827B, ID026A, ID820B).

## 4. Discussion

This study is one of the first attempted to examine both facilitators and barriers to healthy dietary behaviors in aged Chinese-Canadian immigrants using ecosocial theory including personal, familial, community and societal factors.

### 4.1. Personal Factors

The facilitators and barriers found in this study agree with previous studies of psychosocial factors influencing immigrant diet. Across a variety of immigrant backgrounds, minorities tend to have unique cultural beliefs about the foods that constitute a healthy diet [25]. Similarly, a number of participants had personal beliefs on the positive influence that diet can have on overall health status. One previous study also reported the importance of having hope as an intrinsic motivator to pursue a healthier diet [26]. In this study, Asian-American immigrants (*n* = 119) self-reported their dispositional level of goal-directed thinking on the Hope Scale. Asian immigrants who had high levels of hope and knowledge of cardiovascular outcomes were significantly more likely to reduce their salt and fat intake than those who had low levels. Similarly, participants in this study reported that intrinsic motivation was an important facilitator of improving their diet. In this study, some participants were reluctant to consume milk products due to lactose intolerance. In agreement, Chinese-American families tend to consume less milk products than other Americans [27]. However, their reasons tend to be due to unawareness of the importance of consuming milk products for bone health rather than lactose intolerance [27]. These differences in reasoning may be due to the usage of a more open-ended questionnaire, whereas participants in the other study were explicitly asked about their milk consumption.

This study reports some novel findings in regards to personal factors that influence dietary behaviors in aged Chinese-Canadian immigrants. It is indicated that aged immigrants who have a health condition or had a profession in healthcare are more likely to engage in healthy eating, whereas aged immigrants who are difficult to change their eating habits are less likely to do so. Thus, this study showed that aged Chinese immigrants are a unique cohort in that they maintain a mixture of factors including personal motivation, decades of eating habits, and food intolerances that healthcare providers need to be aware of when communicating.

Potential mechanisms to explain the findings may be related to psychological factors including self-efficacy, the high prevalence of lactose intolerance in Asian populations, and the types of foods that encompass the traditional Chinese diet. In Chinese-Americans, self-efficacy and perceived benefits are the most influential psychological factors to improve diet [28]. For example, Chinese-Americans who believed that they had the willpower to maintain small portion sizes and consume an adequate amount of fruits and vegetables were more likely to engage in healthy behaviors [28]. Similarly, Chinese immigrants who are goal-driven and have knowledge of cardiovascular diseases are more likely to consume less salt and fat than those who do not have these personality factors [26]. The low intake of calcium in the participants may be explained by the high prevalence of lactose intolerance in Chinese populations. A recent meta-analysis revealed that about 85% (95% CI, 83–86) of Chinese individuals have lactose malabsorption [29]. Lastly, many of participants expressed that it would be difficult for them to alter decades of cooking tradition that may be high in salt, low in potassium, and low in calcium. The typical Chinese diet consists of wheat, rice and pork [5], which might explain the tendency of Chinese individuals’ imbalances in some nutrients.

### 4.2. Familial Factors

Similar findings have been documented in regards to adults prioritizing children’s desires over parents’ own dietary needs. Some Chinese-American parents reported that they initially resist their children’s desires to eat American food, but eventually oblige, so the entire family eats food that may be unhealthy [30,31]. However, another study reported that the majority of Chinese-American parents do not change their own diet in response to their children’s wishes, as they are accustomed to eating traditional Chinese meals [30]. These differences may be due to differences in the age-range of participants. The age of the parents ranged from 33 to 54 [30], whereas the participants in this study had a mean age of 60.8. Thus, the participants in this study are more likely to be grandparents rather than parents, and may, therefore, respond differently to children’s wants. Many grandparents in China are primary caregivers, and tend to oblige to their grandchildren’s wants by cooking frequent and large meal portions [32]. In addition, grandparents in China tend to cook most family meals and try to accommodate the entire family’s dietary wants [32].

This study indicates a number of other familial facilitators and barriers to healthy eating in aged Chinese-Canadian immigrants. Facilitators including living in a small family, having support from family, and having a family member with a chronic illness are novel findings in this population. In addition, living as a single parent or alone is a novel finding in regards to barriers. These findings have important implications to healthcare providers and family members in intergenerational households.

The traditional Chinese family structure may be the main mechanism that explains why many aged Chinese-Canadians prioritize the needs of their family over themselves. Many aged Chinese-American immigrants tend to speak little English and are dependent on their family for support [33,34]. Many of them live in an extended household and are the caregivers to their grandchildren [34,35]. Consequently, Chinese-American grandparents may assimilate to various facets of American culture in order to identify with their grandchildren [34]. In addition, family conflict during mealtime is a common occurrence in intergenerational households [36], so aged Chinese immigrants may give in to other family members’ dietary wishes to avoid disagreement.

### 4.3. Community Factors

The findings in this study are in accordance with the community factors explored in other studies. Close to 60% of Chinese immigrants reported that they now understand nutritional information better than before they immigrated as a result of detailed information printed on North American food packages [37]. In addition, more than 60% of Chinese immigrants reported that they are consistently exposed to healthy eating educational campaigns through the media and advertisements [37]. Another study reported that many Chinese-American immigrant women receive their health advice from Chinese newspapers, magazines, and television [31]. In addition, many participants reported that they value the advice of their friends and primary care physicians [31]. In fact, many Chinese immigrants visit traditional Chinese medicine practitioners for medical and dietary advice [31,38].

Results from this study also present novel findings in the role of online social media as a facilitator, and the influence of large gatherings with family and friends as a barrier. Some participants mentioned that they discuss their health with friends using social media platforms that are popular amongst the Chinese community, such as WeChat. Overall, findings in this study have important implications on the implementation of future health educational campaigns targeting the Chinese population.

Potential mechanisms to explain the popularity of viewing Chinese dietary educational materials and health information through native social media may be the English language barrier experienced by many aged Chinese immigrants, as well as their beliefs in Traditional Chinese Medicine. Many aged Chinese immigrants speak little English and, therefore, consume Chinese media (such as newspapers, magazines, and television) which may present dietary and health information. In addition, many Chinese immigrants believe in the general ideology of Traditional Chinese Medicine [31,38]. They believe that a healthy diet must be balanced between two opposing forces, “yin” and “yang”, which are the cold/hot forces in food and the body. Potentially, these beliefs may lead aged Chinese immigrants to pursue a balanced diet, and so their beliefs in Traditional Chinese Medicine may facilitate healthy eating.

### 4.4. Societal Factors

Participants in this study reported accessibility and promotion of healthy foods in supermarkets/grocery stores as the only societal facilitator, while frequent dining at restaurants and leading a busy lifestyle were the barriers to healthy eating. These findings are consistent with other studies that demonstrate the type of food stores and restaurants in one’s environment influence food-choices and diet-related health outcomes [39,40]. The majority of Chinese-Canadian immigrants shop for Chinese groceries at least once a week [41]. In addition, this study showed that there is a statistically significant relationship between buying groceries and self-perceived impact of food on health [41]. Low accessibility to grocery stores can lead to poor diet and health outcomes, as Chinese individuals who consume fruits or vegetables fewer than 3 times/day were 1.84 (95% CI  =  1.13, 3.01) times more likely to report poor health than Chinese who consume 5 or more fruits or vegetables/day [42]. In addition, the finding that leading a busy lifestyle is a barrier to healthy eating is corroborated by evidence suggesting that immigrants with busy schedules consume diets high in fat and sugar [43]. Another study reported that Chinese immigrants tend to eat Western food for breakfast and lunch, as it is easier to buy and prepare in comparison to traditional Chinese meals [31].

Potential mechanisms to explain findings in this study may involve low socioeconomic status, busy lifestyles, and acculturation. In this study, 53.3% of participants had an annual family income less than $20,000. Low socioeconomic status is a general barrier to healthy eating [44,45], as it is harder for economically disadvantaged individuals to buy fresh produce on a limited budget. Low socioeconomic status may explain the busy lifestyles experienced by many participants, as they may be holding jobs with long-hours to support their family. Lastly, acculturation may explain the barrier of eating out in restaurants. Due to their time-consuming lifestyles, Chinese immigrants may eat at Western restaurants due to convenience. In fact, for every year increase in US residence, Chinese immigrants become more acculturated and consume more calories, fat, and sugar [46,47]. Many immigrants arrive to their new country healthy and experience a significant decline in their health status upon living in their new country [48,49].

### 4.5. Implications

Healthcare providers should be aware that there are multiple levels of indicators on aged Chinese Canadians’ healthy eating and a comprehensive assessment, including personal, familial, community and societal factors, is needed. On a personal level, cultural beliefs should be valued, personal motivation should be emphasized, decades of eating habits should be recognized, and food intolerances should be accommodated. Healthcare providers need to be aware of the role that aged Chinese-Canadians have in cooking for their families, as they may be sacrificing their own nutritional needs for other family member’s dietary desires. Meanwhile, aged Chinese-Canadians may need to effectively communicate with their family members in order to develop an effective compromise that meets their own dietary needs. There are a variety of family structures which could have different impact on seniors’ health. Thus, individual family assessment should be emphasized in clinical and community health practice. Health education in the community is well-accepted and healthy eating campaign might be needed for aged Chinese Canadians. To facilitate health promotion and prevention of chronic illnesses, health policymakers should consider funding some community health projects using media platforms that are frequented by aged Chinese immigrants.

### 4.6. Strengths and Limitations

This study is the first to examine the facilitators and barriers to dietary behaviours in aged Chinese-Canadians. In addition, this is the first study to examine these factors guided by ecosocial theory, including personal, familial, community, and societal factors. Despite the novelty of findings, the study has limitations. The low sample size of 30 participants may have limited the ability to find other factors that may influence dietary behaviors. In addition, the majority of participants had an annual household income of less than $20,000, so low socioeconomic status may have confounded the results.

## 5. Conclusions

This study revealed that aged Chinese immigrants in Canada experience unique facilitators of and barriers to healthy eating. These facilitators and barriers exist in various ecosocial levels and can be personal, familial, community, or societal factors. Future health educational interventions and patient counselling targeted to aged Chinese-Canadian immigrants should be cognizant of these unique factors that may influence cardiovascular health and other health outcomes.

## Figures and Tables

**Table 1:** Participant Demographic Characteristics.

Variable	Category	*n*	%
Gender	Women	16	53.3
	Men	14	46.7
Chinese lifestyle	Northern Chinese lifestyle	10	33.3
	Southern Chinese lifestyle	20	66.7
Educational level	High school	3	10.0
	Diploma/Certificate	10	33.3
	Bachelor’s degree	13	43.3
	Master/PhD degree	4	13.3
Employment status	Full-time	5	16.7
	Part-time	2	6.7
	Unemployed	3	10.0
	Self-employed	5	16.7
	Retired	15	50.0
Family income	<$20,000	16	53.3
	$20,001 to 50,000	5	16.6
	$50,001 to 80,000	6	20.0
	>$80,000	3	10.0
Marital status	Single	1	3.3
	Married	25	83.3
	Widowed	2	6.7
	Separated	2	6.7
Have a family doctor	Yes	27	90.0
	No	3	10.0
Smoke	Currently smoke.	2	6.7
	Quit now.	4	13.3
	Never	24	80.0
Drinking alcohol	Yes. Occasionally.	12	40.0
	No.	18	60.0
Parents hypertensive	Mother or father hypertensive	13	43.3
	Both parents hypertensive	6	20.0
	No parent hypertensive	8	26.7
	Unknown	3	10.0
Exercises	Sedentary	3	10.0
	Mild activity level	13	43.3
	Moderate activity level	11	36.7
	Heavy activity level	3	10.0
